# A Case of Cavitary Mycobacterium chimaera

**DOI:** 10.7759/cureus.26984

**Published:** 2022-07-18

**Authors:** Bracha Robinson, Moiuz Chaudhri, Jeffrey A Miskoff

**Affiliations:** 1 Internal Medicine, Hackensack Meridian Ocean Medical Center, Brick, USA; 2 Internal Medicine, Critical Care, Sleep Medicine, Jersey Shore University Medical Center, Neptune City, USA; 3 Internal Medicine, Critical Care, Sleep Medicine, Hackensack Meridian Ocean Medical Center, Brick, USA

**Keywords:** lung disease, cardiac bypass, heater-cooler units, nontuberculous mycobacterium, mycobacterium chimaera

## Abstract

Mycobacterium chimaera is a nontuberculous mycobacterium typically associated with heater-cooler units used in cardiac bypass procedures and is usually of low virulence. Here we present a patient with advanced Mycobacterium chimaera infection without typical risk factors.

## Introduction

Mycobacterium chimaera is a nontuberculous Mycobacterium (NTB) of the Mycobacterium avium complex (MAC) that was first described in 2004 [1]; it is most well-known for causing lung disease outbreaks in patients who have undergone cardiac bypass procedures due to exposure to colonized heater-cooler units. Respiratory disease outbreaks are linked to the formation of biofilms in heated stagnant water, which is a favorable environment for the pathogen [2,3]. Other NTB pathogens are associated with patients with underlying pulmonary disease, but this has only been seen in case reports regarding Mycobacterium chimaera specifically [1,4,5].

## Case presentation

A 58-year-old male presented to the emergency department on July 30th, 2019, complaining of cough with yellow sputum, left upper abdominal pain, and worsening fatigue over the last three weeks. He also noted a 20-pound weight loss over the previous two months. He has a history of chronic obstructive pulmonary disease (COPD), hypertension, and intravenous drug use (IVDU). The patient admitted injecting heroin almost daily, worked as an electrician and lived alone. On physical examination, he was noted to have temporal wasting and scattered rhonchi; the abdominal exam was unremarkable. He was afebrile and breathing 90% on room air and had a body mass index (BMI) of 16.

**Figure 1:**
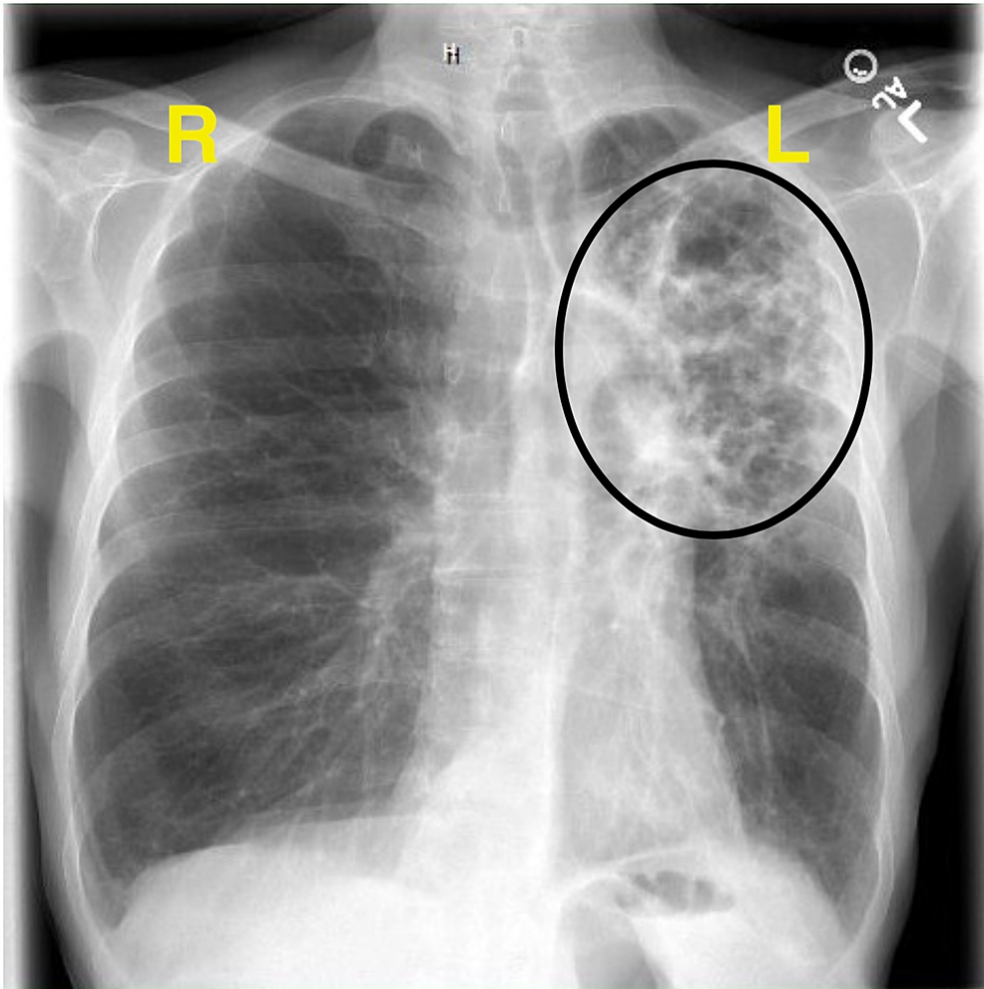
Chest x-ray, posterior-anterior (PA) view, on initial presentation, illustrating left upper lobe interstitial and alveolar process.

He first sought medical attention for his symptoms on May 24th, 2019, when he came to the emergency room complaining of sinus congestion and dyspnea on minimal exertion for one week. At baseline, he could walk one flight of stairs without shortness of breath. He endorsed appetite but denied lower extremity edema, orthopnea, fever, or chills. He was in mild respiratory distress with wheezing and decreased breath sounds at the lung bases on physical examination. On exam, he was afebrile, tachycardic, with a heart rate of 109 beats per minute (bpm), pulse oximetry of 87% on room air, and BMI of 18.5. The emergency physician initially read his chest x-ray as a left upper lobe infiltrate, and he was started on treatment for pneumonia with ceftriaxone, azithromycin, and ipratropium-albuterol nebulizer (Figure 1). The patient subsequently left against medical advice (AMA) and was given a prescription for Levaquin. The radiologist later interpreted the x-ray as a left upper lobe interstitial and alveolar process that may be chronic in etiology. The patient was called back to the emergency room due to positive blood cultures of Candida parapsilosis.

**Figure 2:**
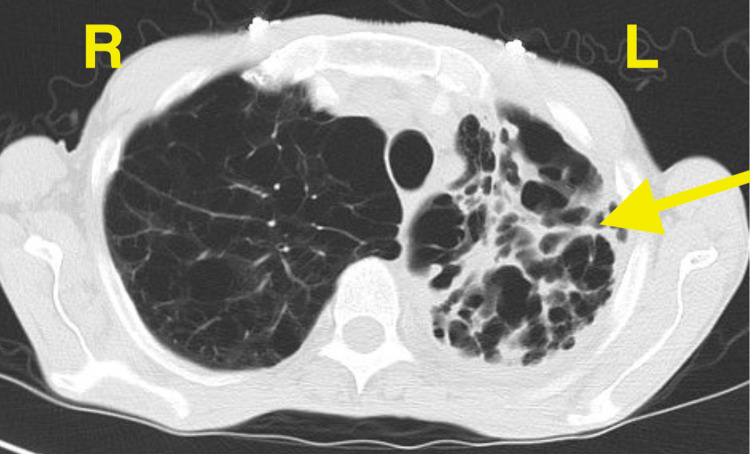
CT scan, transverse view, on initial presentation, illustrating left upper lobe extensive bullous emphysema, bronchiectasis, and cavitations.

Upon his return on May 27th, 2019, he had a computed tomography (CT) scan, which showed left upper lobe extensive bullous emphysema and bronchiectasis (Figure 2). There was also significant volume loss in the left hemithorax, which was suggestive of a chronic process. With his history of COPD and IV drug use, there was suspicion of tuberculosis. While testing was in progress, he was started on fluconazole for candidemia. Endocarditis was ruled out by both a transthoracic as well as by trans-esophageal echocardiogram (TEE). The patient underwent an HIV test, which was negative as well. 

The QuantiFERON®-TB Gold test (QFT-G) (Qiagen, Hilden, Germany) for tuberculosis came back indeterminate, and he was considered to have a low suspicion for tuberculosis. His acid-fast bacilli cultures came back positive, and he was thought to have a MAC infection. After discharge, the cultures were shown to be mycobacterium chimera. The treatment team made the decision not to treat the MAC infection but instead to follow up with the infectious disease and pulmonary specialists in the outpatient setting.

**Figure 3:**
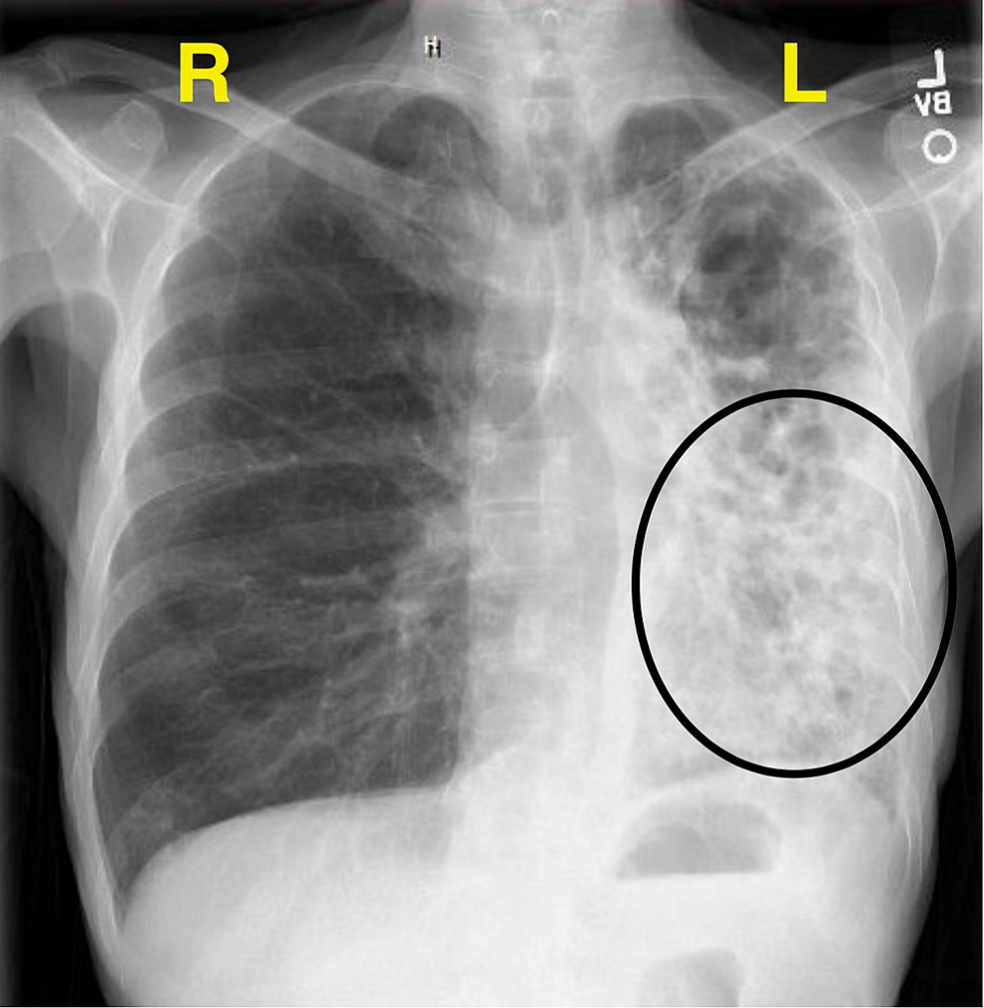
Chest x-ray, posterior-anterior (PA) view, two months later, illustrating increased lung process on the left to now involving the lower lobe.

**Figure 4:**
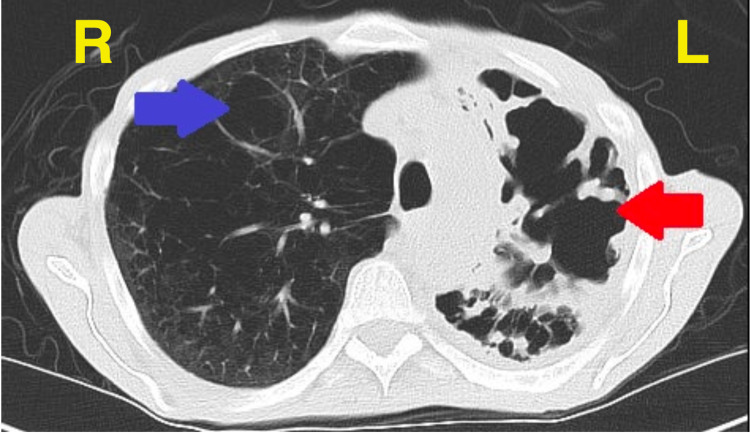
CT scan, transverse view, two months later, illustrating extensive left upper lobe bullous emphysematous changes, including new large bullae (red arrow). The right lung is emphysematous with cavitation (blue arrow).

**Figure 5:**
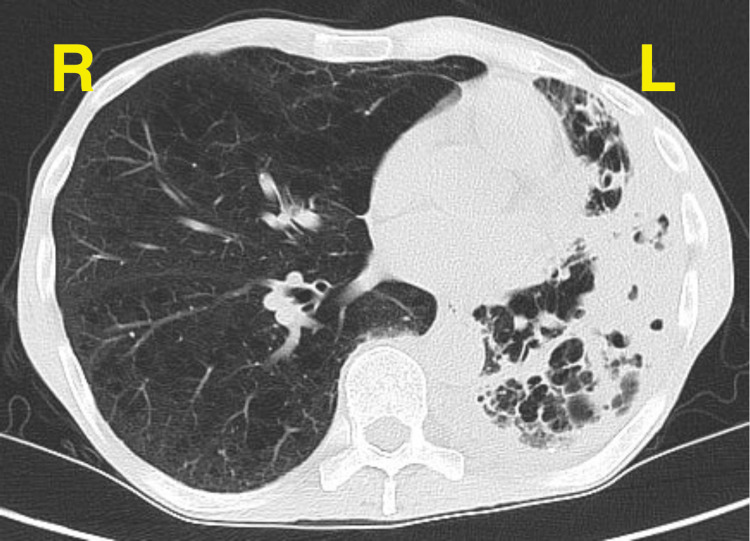
CT scan, transverse view, two months later, illustrating new consolidative changes involving the left mid and lower lungs.

The patient did not follow up in the outpatient setting. Upon his return to the emergency department on July 30th, his imaging showed progression of his Mycobacterium chimaera infection. His chest x-ray showed an increased lung process on the left to now involve the lower lobe (Figure 3). The CT scan showed extensive left upper lobe bullous emphysematous changes. There were new large bullae and new consolidative changes involving the left mid and lower lungs. The right lung was emphysematous with apical scarring (Figure 4, Figure 5). 

At this point, the decision was made to start the patient on a regimen of clarithromycin, ethambutol, and rifabutin. The patient was counseled on the plan to continue these antibiotics for at least a year after discharge.

## Discussion

Mycobacterium avium complex comprises several non-tuberculoid mycobacteria, including Mycobacterium chimaera. This rare pathogen was only recently discovered this century, and treatment guidelines are based on guidelines for other NTB treatments [1]. 

Although it is typically associated with heater-cooler units used in cardiac bypass procedures, a few case reports have described cases of Mycobacterium chimaera in patients with underlying pulmonary disease. A study by Tortoli et al. described a cohort of six patients that included one with underlying bronchiectasis, two with COPD diagnosis, two with pulmonary cavitations, and one without underlying pulmonary disease. Three of the six patients recovered with treatment and without relapse [1]. Bills et al. in 2009 describes a patient with a history of COPD and smoking [4]. A case report by Miskoff et al. in 2018 described one patient presenting with underlying COPD [5]. The reason so few cases of patients with the underlying pulmonary disease have been reported may be due to its recent classification. A study, in 2005, by Lebrun et al., describes several cases of patients with a history of bronchiectasis and lung cancer, which likely had Mycobacterium chimera, but without the confirmatory testing that we have available today [6]. 

The treatment for Mycobacterium avium complex is based on the severity of the disease. For this patient, the decision initially was not to treat due to lengthy treatment duration with drugs that may be difficult to tolerate. For many patients, close follow-up rather than antibiotic therapy is the best course. However, the patient did not follow up in the outpatient setting, and when he returned to the ED, he was found to have advanced disease. For patients with severe disease, it is recommended to take azithromycin, rifampin, and ethambutol daily. For patients with mild to moderate disease, these antimicrobials can instead be taken three times weekly. The duration of therapy is at least 12 months [7-9]. The Infectious Diseases Society of America (IDSA) released an updated guideline on MAC in 2020 which recommended using a more aggressive treatment approach rather than a watch and wait approach in patients with the cavitary manifestation of MAC [10]. 

A novel finding, in this case, is that the patient had positive blood cultures for Candida parapsilosis on presentation. Several studies have shown an association between Mycobacterium tuberculosis and respiratory candidiasis; in one study, the prevalence was as high as 40% [11]. However, the prevalence of co-infections of MAC and candidemia have not been well studied. Candidemia in this patient may be related to his active IV drug use, as there is a known association between IV drug use and candidemia [10]. It is unclear what role his IV drug use played in this case, and it is a potential area of future study.

## Conclusions

Here we presented a patient who developed advanced pulmonary Mycobacterium chimaera infection without typical risk factors of the prior cardiac bypass procedure. Instead, his past medical history was significant for COPD and IV drug use. The decision was initially made for this patient to proceed with close follow-up and only started treatment when his disease progressed. The therapeutic regimen involves clarithromycin or azithromycin and rifampin with ethambutol for a prolonged period, often for at least 12 months. There is a high degree of drug-drug interactions and toxicity. The treating physician can follow the patient in the outpatient setting closely and start treatment when the disease progresses. Mycobacterium chimaera infections should be considered in patients with a history of COPD. The association with IV drug use is a potential area for future study. 
